# The application of optical technology in the diagnosis and therapy of oxidative stress-mediated hepatic ischemia-reperfusion injury

**DOI:** 10.3389/fbioe.2023.1133039

**Published:** 2023-02-20

**Authors:** Lijuan Wang, Jiali Shao, Chen Su, Jinfeng Yang

**Affiliations:** ^1^ Department of Medicine, Hengyang Medical School, University of South China, Hengyang, China; ^2^ Department of Anesthesiology, Hunan Cancer Hospital, The Affiliated Cancer Hospital of Xiangya School of Medicine, Central South University, Changsha, Hunan, China

**Keywords:** optical technology, oxidative stress, hepatic ischemia-reperfusion injury, diagnosis, treatment

## Abstract

Hepatic ischemia-reperfusion injury (HIRI) is defined as liver tissue damage and cell death caused by reperfusion during liver transplantation or hepatectomy. Oxidative stress is one of the important mechanisms of HIRI. Studies have shown that the incidence of HIRI is very high, however, the number of patients who can get timely and efficient treatment is small. The reason is not hard to explain that invasive ways of detection and lack of timely of diagnostic methods. Hence, a new detection method is urgently needed in clinic application. Reactive oxygen species (ROS), which are markers of oxidative stress in the liver, could be detected by optical imaging and offer timely and effective non-invasive diagnosis and monitoring. Optical imaging could become the most potential tool of diagnosis of HIRI in the future. In addition, optical technology can also be used in disease treatment. It found that optical therapy has the function of anti-oxidative stress. Consequently, it has possibility to treat HIRI caused by oxidative stress. In this review, we mainly summarized the application and prospect of optical techniques in oxidative stress-induced by HIRI.

## 1 Introduction

Hepatic ischemia-reperfusion injury (HIRI) refers to the condition that the ischemic liver is further damaged by blocking the hepatic hilum and reperfusion during liver surgeries such as liver resection, or transplantation (Cannistrà et al., 2016). Hepatectomy and liver transplantation are the two major surgical procedures for the treatment of primary liver cancer, which is the sixth most common cancer in the world (Sung et al., 2021; Maki and Hasegawa, 2022). HIRI is generally unavoidable during both operations (Sun et al., 2022). HIRI is the most common cause of hepatic dysfunction or functional failure after liver surgery and can even be life-threatening (Lentsch et al., 2000). Study has shown that HIRI could cause 10% of the early graft failure in liver transplantation surgery (Uchida et al., 2010). How to recognize HIRI happened and supply efficient treatments are urgently needed. However, the mechanisms of HIRI are very complex. The direct injury of hepatocytes by hepatic ischemia is induced by hypoxia. And during reperfusion, oxidative stress (OS) and calcium overload damage the hepatic sinus endothelial cells, thereby causing secondary hypoxic reoxygenation damage (Piper et al., 1996; Montalvo-Jave et al., 2008). In addition, hepatic portal occlusion could cause intestinal blood stasis, increase the release of endotoxin in the blood, pro-inflammatory cytokines, chemical factors, protease, activation of Kupffer cells and accumulation of neutrophil, which lead to secondary immunogenic injury (Guan et al., 2014; Oliveira et al., 2018). These mechanisms exacerbate hepatocellular death and may lead to liver dysfunction. Unfortunately, there is currently a lack of clinically effective means to diagnose HIRI in a timely manner during the operation and no approved drugs for the intervention of HIRI. Pathological examination and biochemical examination are two gold standard methods widely used in current HIRI diagnosis (Arab et al., 2009). However, the disadvantage of pathological examination is that it is invasive and biochemical examination cannot provide morphological and pathological information. A new non-invasive quantitative method is urgently needed. Oxidative stress is caused by excessive production of reactive oxygen species (ROS), which could be imaging *in vivo*. Oxidative stress is considered as one of the major causes of ischemia-reperfusion injury, and this response involves both direct and indirect cytotoxic mechanisms (Lentsch et al., 2000; Abu-Amara et al., 2010; Monga, 2018). Imaging of oxidative stress is important for the study of the mechanisms, even for diagnosis and treatment of HIRI. Studies have shown that inhibition of oxidative stress can alleviate hepatic ischemia-reperfusion injury (Galaris et al., 2006; Jaeschke and Woolbright, 2012; Elias-Miró et al., 2013; Guan et al., 2014).

Currently, the explosive development of optical imaging provides a new and feasible opportunity for further research on the molecular mechanism of HIRI and for the timely, effective diagnosis. The increased amount of ROS produced in HIRI could be imaged by optical imaging techniques such as fluorescence imaging (FI) and photoacoustic imaging (PAI) (Bai et al., 2019; Chen et al., 2021). Optical imaging can visualize physiological and pathophysiological processes at the cellular and molecular levels *in vitro* and *in vivo* with advantages of real-time, effective, specific and non-invasive detection of ROS(Pirovano et al., 2020, Müller et al., 2013). Based on these advantages, optical imaging could be identified as an important option for the efficient diagnosis of HIRI.

Additionally, optical technology also holds promise for clinical treatment of diseases. Light therapy is a method of using sunlight or artificial light (infrared, ultraviolet, visible, laser) to promote the recovery of the body and cure diseases (Xu et al., 2020; Johnson et al., 2022). The common light treatments, including photodynamic therapy (PDT), photobiomodulation therapy (PBMT), and laser therapy (LT), have attracted the attention of researchers and were tried to apply in clinic in recent years for advantages of accurate spatial localization, rapid optical response, suitable penetration depth, simple operation and non-invasive, and so on (Zhi et al., 2020; Glass, 2021; da Silva et al., 2010; Song et al., 2022a). PBMT and low-intensity laser therapy (LILT) have been shown to have antioxidant properties, providing new therapeutic directions for oxidative stress-HIRI (Takhtfooladi et al., 2014; Leal-Junior et al., 2019; Mansouri et al., 2020).

In this review, we briefly describe the pathogenesis of HIRI and the possible mechanisms of oxidative stress. Then, we summarize the application of optical imaging methods and list some special fluorescent probes for oxidative stress. At the last, we focus on the way that optical imaging assists the diagnosis of HIRI by detecting reactive oxygen species, and light therapy inhibits ROS-mediated tissue damage to treat HIRI.

## 2 The most widely accepted mechanism of HIRI: Oxidative stress

**FIGURE 1:**
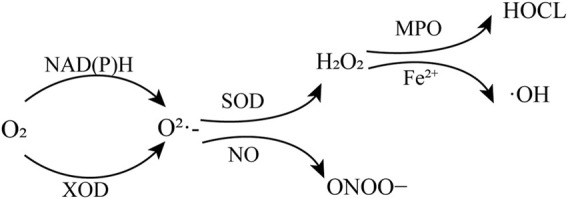
Production of reactive oxygen species. NAD(P)H, nicotinamide adenine dinucleotide and nicotinamide adenine dinucleotide phosphate; XOD, oxidase and xanthine oxidase; SOD, superoxide dismutase; MPO, myeloperoxidase.

Oxidative stress has been considered as one of the main risk factors in reperfusion injury. Many highly reactive molecules, ROS, are generated during the period of HIRI to induce oxidative stress (Jaeschke and Woolbright, 2012; Cannistrà et al., 2016). ROS include superoxide anion (O_2_·^-^), hydroxyl radicals (·OH), hydrogen peroxide (H_2_O_2_), peroxynitrite (ONOO−), and hypochlorous acid (HOCL) (Bai et al., 2019). During reperfusion, restoration of blood flow in the ischemic liver induces the overproduction of superoxide anion (O_2_·^-^) through enzymatic pathways such as nicotinamide adenine dinucleotide phosphate (NADPH) oxidase and xanthine oxidase (XOD) catalysis (Jaeschke and Woolbright, 2012). Then O_2_·^-^ is further converted into H_2_O_2_ by superoxide dismutase (SOD) (Galaris et al., 2006). H_2_O_2_ reacts with chlorine ions catalyzed by myeloperoxidase (MPO) to produce the much more potent oxidant HOCL. Or in the presence of transition metal ions, such as Fe^2+^, H_2_O_2_ can form hydroxyl radicals (·OH) *via* Fenton reaction (Gaut et al., 2001; Du et al., 2015). Another interaction of O_2_·^-^ is with nitric oxide (NO) to generate ONOO^−^, which is a strong oxidant and nitrating agent (Bai et al., 2019) (Figure 1). The above reactive oxygen species could oxidize DNA, proteins and lipids to cause a range of harmful cellular reactions, including inflammation, organelle damage and cell death (Galaris et al., 2006; Huang et al., 2022).

Studies have shown that the Kupffer cells (KCs) in the liver represent are one of the major sources of ROS and inflammatory mediators during HIRI (Rymsa et al., 1991; Jaeschke et al., 1993; Togashi et al., 2000). In the initial phase of reperfusion (less than 2 h after reperfusion), KCs could be activated by tumor necrosis factor (TNF-a), interleukin (IL)-1 and other factors, which are related with liver injury during ischemia or produced immediately after reperfusion with reoxygenation (Serracino-Inglott et al., 2001; Galaris et al., 2006). NADPH oxidase is the main source of superoxide formation of Kupffer cells (Luangmonkong et al., 2018).

In addition, active and pro-inflammatory cytokines produced during ischemia and reperfusion could bind to neutrophil and lymphocyte receptors, recruiting and activating neutrophil (Martinez-Mier et al., 2000). Neutrophil NADPH oxidase, specifically NOX2, is also the main source of ROS. Upon activation, neutrophils produce O_2_·^-^ through NADPH oxidase, then generate H_2_O_2_, ·OH, and HOCL together with proteases (El-Benna et al., 2008). And the neutrophil could release MPO that convert H_2_O_2_ into HOCL, a powerful oxidant (Guan et al., 2014). The neutrophil-mediated oxidative stress was mainly in the late stage of reperfusion (within 48 h of reperfusion) (Hasegawa et al., 2005; Galaris et al., 2006).

Another critical source of ROS is xanthine oxidase (XO) (Fernández et al., 2002). However, this statement lacks direct evidence, supported only by the protective effect of the xanthine oxidase inhibitor allopurinol on HIRI and indirect evidence based on OS (Nordström et al., 1985; Metzger et al., 1988). Other sources of ROS include mitochondria, non-phagocytic NADPH oxidases, and others (Li and Shah, 2001; Murphy, 2009).

The main mechanisms of oxidative stress-induced liver injury include lipid peroxidation (LPO), the mitochondrial membrane permeability transition (MPT), apoptosis and necrosis (Lemasters et al., 2002; Negre-Salvayre et al., 2010; Jaeschke and Woolbright, 2012; Li et al., 2015). That is to say, large amounts of ROS are produced in the early stages of HIRI by pathways such as neutrophil, Kupffer cells or XO. The increase of ROS production leads to increased cell death through necrosis or apoptosis. The apoptotic and necrotic cells in turn cause the aggregation of Kupffer cells and neutrophil cells, further damaging the cells and leading to the continuous production of ROS during reperfusion, which then forms a vicious cycle.

## 3 The application of optical imaging technologies with oxidative stress-mediated HIRI

At present, the relevant molecular mechanisms of hepatic ischemia-reperfusion injury have been widely studied, but due to the lack of real-time, effective and specific diagnostic methods, how to prevent and treat HIRI is still a thorny clinical problem. Pathological examination and biochemical examination are two gold standard methods widely used in current HIRI diagnosis (Arab et al., 2009). However, the disadvantage of pathological examination is invasive and biochemical examination cannot provide morphological and pathological information. Optical imaging technology refers to the method that combines optical detection means with optical detection molecules to image cells or tissues or even organisms to obtain biological information, including fluorescence imaging (FI), bioluminescence imaging (BLI), optoacoustic imaging (OAI) and Optical Coherence tomography (OCT) and et al. (Pirovano et al., 2020). Optical imaging has become important tools in biomedicine because of non-invasive visualization of physiological and pathophysiological processes at the cellular and molecular level *in vivo* with a high degree of specificity and real-time, as demonstrated in different animal disease models (Müller et al., 2013; Pirovano et al., 2020). Optical imaging technology of HIRI can quickly detect the molecular and cellular processes in the process of HIRI, which is a method to solve the above problems. It can provide accurate information for the early diagnosis of HIRI and monitor the treatment process in real time. Optical imaging technology based on oxidative stress has achieved some achievements in the exploration and research of diagnosing HIRI at present. To date, various optical imaging techniques have been developed for clinical diagnosis. For example, photoacoustic imaging is used in the diagnosis of breast cancer and skin cancer, and autofluorescence imaging is used in the clinical diagnosis of ophthalmic diseases (Heijblom et al., 2016; Yung et al., 2016; Chen et al., 2017). In this review, we list some relevant studies on optical imaging in the diagnosis of oxidative stress-mediated HIRI.

### 3.1 Bioluminescence imaging (BLI)

The principle of BLI is that luciferase, which is artificially injected, catalyzes the oxidation of its substrate luciferin to emit light (Müller et al., 2013). BLI does not require any external excitation light source with the advantage of a lower background. Therefore, very low levels of the target molecule could be detected (Dothager et al., 2009). However, luciferase is a naturally occurring enzyme in insects, mainly found in firefly, sea pansy, and green or red click beetles (de Wet et al., 1987; Bhaumik and Gambhir, 2002; Miloud et al., 2007). The application of BLI requires a genetic alteration of the target tissue to achieve the expression of the luciferase gene in cells (Michelini et al., 2009). Consequently, BLI is difficult to apply in clinical settings, but in animal models. In addition, the limited application of BLI *in vivo* is also related to the low tissue penetration depth caused by the short wavelength light emitted by fluorescein (Sato et al., 2004). Herein, we mainly describe the application of FI and OAI for oxidative stress.

### 3.2 Fluorescence imaging (FI)

The principle of FI is that the fluorescent material is excited by a specific external energy (such as high-energy rays or laser), causing its electron orbit to transition to high-energy orbit, the fluorescence signal can be detected when the energy is released to the ground state (Müller et al., 2013). The fluorescent groups in common use at present include a variety of small molecule fluorescent dyes, green fluorescent protein and red fluorescent protein, quantum dots (QDs), and up-conversion luminescent materials. The main advantage of fluorescence imaging is its high sensitivity, in addition to non-invasive. Very small amounts of imaging agents (nanometer to femtometer or less) can be detected (Müller et al., 2013). But, the main limitation of FI is that due to the absorption and scattering of light by biological tissues and body fluids, non-specific light absorption limits the depth of penetration to a few millimeters, which makes florescence imaging only suitable for superficial targets or body regions of limited size in clinical (Wang et al., 2022a). However, because of low phototoxicity to cells, minimal interference to hemoglobin absorption, low autofluorescence and good tissue penetration of near-infrared (NIR) fluorescence, whose absorption and emission maximums are in the near-infrared region (650-900 nm), near-infrared (NIR) fluorescence imaging is more suitable for tissue and individual imaging. Therefore, NIR FI has more advantages in surgical imaging (Weissleder and Ntziachristos, 2003; Wang et al., 2022a). At present, fluorescence imaging is commonly used to detect markers of oxidative stress in optical imaging. There have been a large number of fluorescent probes. Dichlorodihydrofluorescein (DCFH_2_), hydroethidine (HE), and MitoSOX™ Red are the most widely used fluorescent probes to monitor H_2_O_2_, O_2_·^-^ and other ROS by far. But these are considered to be the starting points for further research (Bai et al., 2019). On the basis of these probes, researchers synthesized ROS sensors with different characteristics to better explore the relevant mechanisms of ROS in disease process, disease diagnosis and intervention.

**FIGURE 2:**
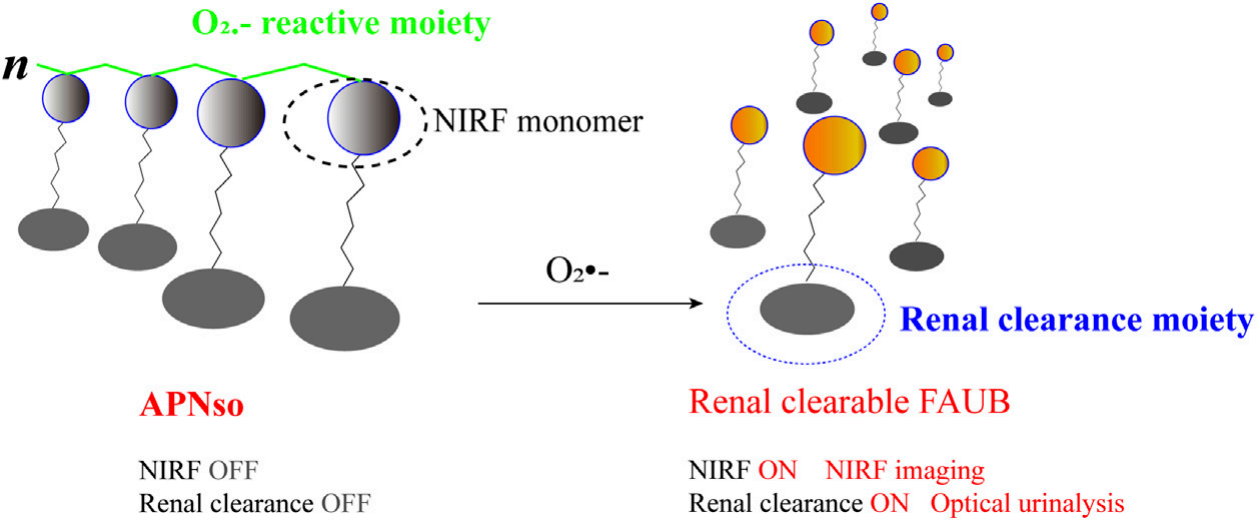
Schematic representation of the APN_SO_ probe for HIRI NIRF imaging and urine analysis.

For example, the results of the study by (Huang et al. (2022) shows that the probe, named APN_SO,_ could detect the change of O_2_·^-^ in animal model with HIRI to diagnosis HIRI and evaluate the therapeutic effect. Upregulated O_2_·^-^ during HIRI could cleave trifluoromethanesulfonate of APN_SO_ probes and induce self-elimination to depolymerize the backbone of APN_SO_. Then, a fluorescent artificial urinary biomarker (FAUB), fragment of a fluorophore that can be cleared by the kidney, is released for non-invasive *in vivo* imaging and urine analysis of HIRI (Figure 2). One of advantages of this probe is that real-time NIR fluorescence imaging of oxidative stress during HIRI with systemic administration of APN_SO_ is found to detect hepatic IRI at least 7 h earlier than serum ALT/AST and histological assays. Another advantage is APN_SO_ allows remote detection of liver IRI by *in vitro* urine analysis. The effectiveness and safety of the probe have been demonstrated in animal models of HIRI. In the near future, further verification of it in clinic may provide new hope for the early diagnosis of HIRI. Some researchers also have constructed a two-photon excitation-ratio fluorescence probe, which could be targeted to image the O_2_·^-^ in mitochondrial. This study reveals a possible transport pathway of mitochondrial O_2_·^-^ during HIRI, and might provide a new strategy and approach for HIRI diagnosis and therapy (Zhang et al., 2019).

**FIGURE 3:**
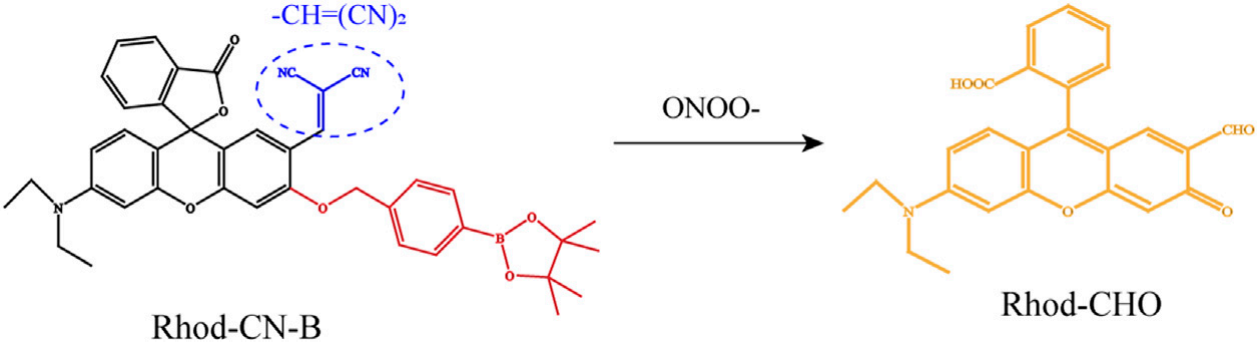
Schematic representation of the chemical structure of the Rhod-CN-B probe responding to ONOO−. Adapted with permission from (Peng et al., 2022). Copyright © 2022. American Chemical Society.

In addition, elevated ONOO− levels were proved to be associated with aggravation of hepatocyte injury. Accordingly, we and our co-authors report that the probe, named Rhod-CN-B modified by a strong electron-withdrawing methylene malononitrile functional group [−CH=(CN)_2_] at the 2’position of Rhodol-based dyes could detect ONOO- without interference of other ROS, such as HOCL, H_2_O_2_ showing high signal-to-noise ratio, good selectivity, photostability and fast response (within 10 s) (Figure 3) (Peng et al., 2022). *In vitro*, FI with Rhod-CN-B probe of generation of ONOO− are successfully achieved during the LPS-induced cell apoptosis process. *In vivo*, Rhod-CN-B could be applied to detect fluctuations of ONOO−, which is proved to have high levels in animal model of HIRI. Herein, we only review two characteristic reactive oxygen species-based optical imaging of HIRI. There is much more to the story than that. In Table1, we briefly introduce the basic information and characteristics of some other common and characteristic probes for fluorescence imaging.

**TABLE 1:** Summary of optical imaging probes and their application for the oxidative stress.

Sensor	Optical imaging	Types	Characteristic	Application	References
TCA	Dual-mode imaging (combine the merits of One-photon and two-photon fluorescence imaging	O_2_•^−^	TCA exhibits high selectivity for O_2_•^−^ and has reversibility mediated by O_2_•^−^ or glutathione (GSH)	Hepatocytes	Zhang et al. (2013)
				Zebrafish and mice with IR injury	
CST	Fluorescence imaging	O_2_•^−^	Quantitative real-time imaging of mitochondrial O_2_•^−^	HIRI in mice	Zhang et al. (2019)
			Reveal relevant molecular mechanisms of OS in HIRI		
CPR-SK	Quantitative fluorescence imaging	O_2_•^-^Keap1	Permit *in situ* synergistic imaging of O_2_•^−^ and Keap1 and reveal the OS related molecular mechanisms of HIRI	Human hepatocyte IRI model	Zhang et al. (2022)
REPOMS	NIR-IIb fluorescent redox probe	•OH	REPOMs is a reversible probe that could respond to the ROS/GSH cycles to show characteristics in monitoring the ROS fluctuations during HIRI in real time both *in vitro* and *in vivo*	HepG2 cells	Song et al. (2022b)
		GSH		Mouse with HIRI	
RTFP	Fluorescence microscopic imaging	ONOO-	Visualization of ONOO- level without interference from other biomolecules	Drug-induced liver injury	Cheng et al. (2020)
				Non-alcoholic fatty liver disease	
CyCA	Fluorescence imaging	ONOO−	Enable real-time *in situ* visualize O_2_•^−^ and ONOO− changes in mitochondria and find direct molecular links of O_2_•^−^/ONOO−/arginase 1 in HIRI	Ischemia-reperfusion models in cells and mice	Zhang et al. (2019)
		O_2_•^−^			

The detection of specific autofluorescence (AF) by fluorescence lifetime imaging (FLIM) may also be an effective diagnostic method for hepatic ischemia-reperfusion injury. Fluorescence lifetime imaging is a kind of FI. The brightness of the pixels in the resulting image represents fluorescence lifetime, not fluorescence intensity. Fluorescence lifetime refers to the average time that a molecule of a fluorescent substance is in an excited state after it has absorbed photons (Alfonso-Garcia et al., 2021). Common AFs within liver tissue include NAD(P)H, flavin, lipofuscin, lipofuscin-like lipoprotein, and, retinoid, porphyrin, bilirubin and so on (Croce et al., 2018). Among them, NAD(P)H and flavin are coenzymes participating in reductive biosynthesis and antioxidant defense and used for the *in situ* or *in vivo*, real-time monitoring of organ energy state and response to ischemia/reoxygenation (Heikal, 2010). Some researchers have imaged HIRI mice *in vivo* by FLIM without routine biopsy or fluorescent dye (Thorling et al., 2013; Wang et al., 2015). Besides, lipofuscins and lipofuscin-like lipoproteins have recently been regarded as biomarkers of oxidative stress in the liver tissue. Excessive lipid oxidation caused by oxidative stress could lead to the accumulation of lipofuscins and lipofuscin-like lipoproteins, which could emit high degree of fluorescence during HIRI (Seehafer and Pearce, 2006). The *in situ* optical detection of lipofuscins and lipofuscin products could also play an important role in improving the real-time monitoring of oxidative stress and the diagnosis of hepatic ischemia-reperfusion injury.

### 3.3 Optoacoustic imaging (OAI) or photoacoustic imaging (PAI)

Optoacoustic imaging (OAI), also known as photoacoustic imaging (PAI), is a new technology that combines light excitation with ultrasound detection for biomedical imaging (Deán-Ben and Razansky, 2021). The principle of photoacoustic imaging is when a laser irradiates tissue, the light absorbers in biological tissues absorb energy and convert it into heat energy, the heat expansion and cold contraction of the absorbers make them become sound sources, and the ultrasonic transducers located around the tissues acquire the photoacoustic waves generated, and through signal processing and photoacoustic image reconstruction, the photoacoustic images reflecting the internal structure and function of the tissues are formed (Pirovano et al., 2020; Glatz et al., 2011; Tzoumas and Ntziachristos, 2017). OAI combines the advantages of high sensitivity and resolution of optical imaging with the advantages of ultrasonic imaging, which can image tissues several centimeters deep. At the same time, OAI can improves the drawbacks of depth limitations of conventional fluorescence imaging and shortness of poor contrast of ultrasound imaging. Finally, it can realize real-time non-destructive imaging of deep tissue with high resolution, high contrast and high penetration depth. And, multi-spectral photoacoustic tomography (MSOT) technology, realizing spectral mixing, and raster-scan optoacoustic mesoscopic imaging (RSOM) technology, which can carry out multi-band splitting, generate more details in disease-related imaging, affording high-resolution optoacoustic imaging for cellular, tissue and whole-body resolution (Omar et al., 2015; Johnson et al., 2019). In clinical, photoacoustic imaging has been used to imaging Crohn’s disease, breast cancer, and skin cancer (Heijblom et al., 2016; Chen et al., 2017; Knieling et al., 2017; Deán-Ben and Razansky, 2021).

**FIGURE 4:**
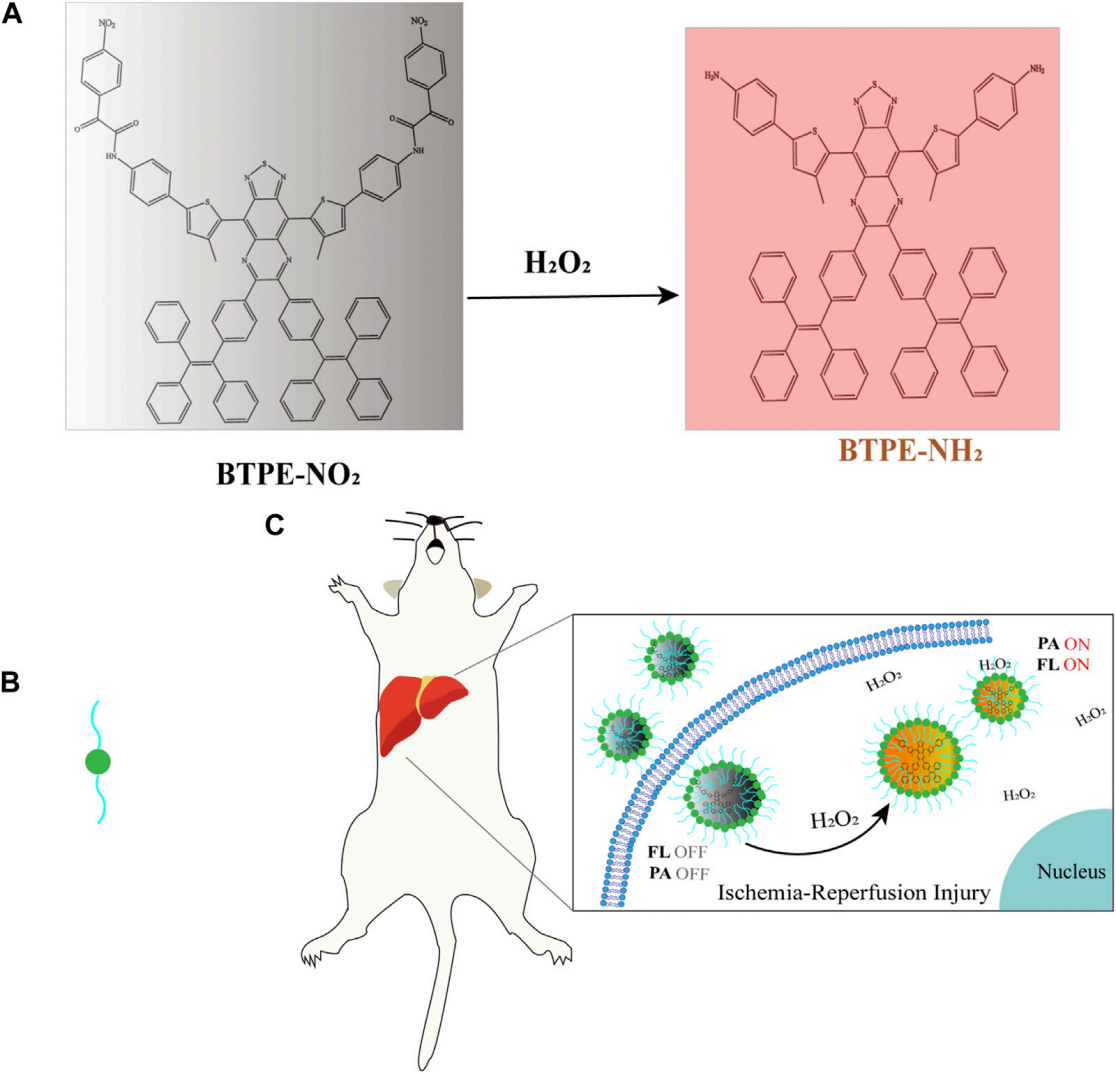
**(A)** The structure and luminescence mechanism of BTPE-NO_2_@F127 (Chen et al., 2021). **(B)** Represent the amphiphilic and biocompatible polymer Pluronic F127. **(C)** The H_2_O_2_-activating probe BTPENO_2_@F127 was used to image hepatic I/R injury in a mouse model.

OAI can also be used to image the changes of ROS to diagnose and monitor ROS-related diseases. For example, fluorescence/photoacoustic (FL/PA) bimodal imaging of excess H_2_O_2_ produced *in vivo* can be performed by probes, TPP-HCy-BOH and BTPE-NO_2_@F127, to diagnose pathologic inflammation (Chen et al., 2020; Chen et al., 2021). The probe, BTPE-NO_2_@F127, has been validated in a mouse model with HIRI. Here we illustrate the application of OAI in the diagnosis of HIRI with this probe. The probe BTPE-NO_2_@F127 includes three parts. The first part is a benzothiadiazole-based core, which is synthesized by combining benzothiadiazole with two hydrophobic molecular rotors of tetraphenyl ethylene (TPE) to make the activated chromophore BTPE-NH_2_ have AIE activity and enhance the aggregation degree. Second part is two nitrophenyloxy acetamide groups which are added at both ends of the benzothiadiazole core to serve as the identification part of the biomarker H_2_O_2_ and the emission quenching agent for their electron-withdrawing ability. Third, the amphiphilic and biocompatible polymer Pluronic F127 was used to encapsulate the BTPE-NO_2_ molecule to make sure the probe has the necessary biocompatibility and water-dispersibility. The pathological level H_2_O_2_ at the liver with ischemia-reperfusion injury cleaves nitrophenyloxy acetamide and then produces BTPE-NH_2,_ which could absorb light at 680–850 nm and result in enhanced NIR-II fluorescence and photoacoustic intensity (Figure 4). Thereby, the nanoprobe, BTPENO_2_@F127, could detect, image and diagnosis the hepatic ischemic-reperfusion injury with OAI and NIR-II fluorescence imaging by responding to H_2_O_2_ which is biomarker of oxidative stress. And, in regard of the efficacy and safety of this probe, the authors have validated in a mouse model of hepatic ischemia/reperfusion injury (Chen et al., 2021). But whether the fluorescence imaging of this probe could be used in clinic need to be further explored.

## 4 Light therapy based on oxidative stress-mediated HIRI

Various therapeutic strategies have been developed for the pathogenic mechanisms of HIRI to facilitate the progression of drugs to mitigate HIRI. Because of high ROS levels are one of the main pathogenic factors of HIRI, one of the current pharmacological treatment strategies is to focus on targeting ROS production to alleviate HIRI. A large number of studies have found that anti-oxidative stress is effective in the treatment of hepatic ischemia-reperfusion injury (Galaris et al., 2006; Jaeschke and Woolbright, 2012; Guan et al., 2014). One of the common approaches to reduce HIRI may be ischemic preconditioning, whereby the production of antioxidants (such as SOD, NO), which are induced by transient I/R prior to long-term hepatic ischemia (Carini and Albano, 2003; Massip-Salcedo et al., 2007; de Rougemont et al., 2009). And, antioxidant enzymes, miscellaneous antioxidants or vitamins E and C are used to inhibit the formation of ROS or scavenge reactive species to protect liver injury in clinical (Galaris et al., 2006). However, because of the short blood circulation time and serious side effects of these drugs, the clinical effect is not good (Liu and Grodzinski, 2021). In recent years, with the development of optical technology, it is gradually moving towards medicine. Light therapy, mainly including photodynamic therapy (PDT), photothermal therapy (PTT), low-intensity laser therapy (LILT) and photobiomodulation therapy (PBMT), is a method of using artificial light including infrared ray, ultraviolet ray, visible light, or laser to prevent and cure diseases with advantages of accurate spatial localization, rapid optical response, suitable penetration depth, simple operation and non-invasive, and so on (Xu et al., 2020; Johnson et al., 2022). The anti-oxidative stress mechanism of optical therapy provides a new possibility and treatment direction for HIRI induced by oxidative stress.

### 4.1 Photodynamic therapy (PDT) and photothermal therapy (PTT)

With the development of optical technology, optical therapy has been a clinical option for the treatment of some diseases. For example, photodynamic therapy (PDT) and photothermal therapy (PTT) are promising approaches to cancer therapy. PTT is a new method of non-invasive tumor therapy, which can transform light energy into heat energy to kill tumor cells by using photothermal agent (PTA) under the irradiation of NIR and other external light sources (Zhao et al., 2021). The three main mechanisms of anti-tumor effects of PDT are: 1) Direct cytotoxicity, 2) destruction of tumor vessels, and 3) stimulation of anti-tumor immunity which contributes to be immunologically silent or even immunosuppressive (Dąbrowski and Arnaut, 2015). Besides, this kind of therapy is to kill tumor cells by activating oxidative stress and exerting the cytotoxic effect of oxidative stress (Donohoe et al., 2019). The formation of ROS during PDT occurs when tissue-absorbed photosensitizers are excited by a specific wavelength of laser irradiation, and the excited photosensitizers transmit energy to the surrounding oxygen, producing highly reactive singlet oxygen and other ROS (Donohoe et al., 2019). However, HIRI requires inhibition of oxidative stress and reduction of ROS production to alleviate hepatic injury. So, PDT and PTT may be not suitable for the treatment of HIRI.

### 4.2 Low-intensity laser therapy (LILT)

Low-intensity laser therapy (LILT) is a treatment that uses low-power lasers with the range of 1–1,000 mW and at wavelengths from 632 to 1,064 nm to stimulate biological responses. The advantages of LILT are no heat, sound and vibration (Takhtfooladi et al., 2014). Currently, LILT is used clinically in dentistry, musculoskeletal disorders, and others for its role in promoting wound healing and relieving pain (Glazov et al., 2016; Baxter et al., 2017; Clijsen et al., 2017; Ren et al., 2017; Nadhreen et al., 2019). In addition, LILT can suppress OS, providing the possibility for the treatment of HIRI. The mechanism is that the cellular chromophores or photoreceptors in the mitochondria, when exposed to low-intensity laser light, affect mitochondrial respiratory chain processes, ultimately leading to increased production of adenosine triphosphate (ATP), ROS, and the release or production of NO (Chung et al., 2012). And at present, the application of low-intensity laser therapy in liver diseases has been studied experimentally. Some researchers have found that LILT can improve liver cirrhosis induced by carbon tetrachloride (CCl_4_) in animal models (Oliveira-Junior et al., 2013). Besides, prophylactic use of laser therapy before ischemia can restore mitochondrial function and fatty acid binding protein expression to alleviate ischemic injury (Vilalva et al., 2018). Takhtfooladi et al. (2014) showed that LILT which is applied in transcutaneous manner could effectively improve HIRI in rat models. In this study, LILT may protect the liver injury through antioxidation, for changing GSH and MDA levels in rats with HIRI. But other than that, LILT after acute hepatectomy can also significantly enhance regeneration of liver (Oron et al., 2010).

In conclusion, LILT is a potential treatment for HIRI. However, the application of LILT in HIRI therapy remains at the laboratory level. The clinical transformation of LILT still needs more researches.

### 4.3 Photobiomodulation therapy (PBMT)

The photobiomodulation therapy (PBMT) is a new type of optical therapy, in which the emitted visible to infrared broadband light through light sources, such as lasers, light-emitting diodes (LEDs), interacts with the chromophore and triggers chemical and physical responses in tissues (Leal-Junior et al., 2019). Studies have proved that PBMT could decrease oxidative stress to make a difference (De Marchi et al., 2012). Dos Santos et al. (2017) think that PBMT improves mitochondrial function to decrease formation of ONOO− . PBMT could also reduce generation of H_2_O_2_ *via* catalase (CAT) and glutathione peroxidase (GPX) (Ferraresi et al., 2012). Brain photobiomodulation (PBM) therapy plays a therapeutic role in dementia and Parkinson’s disease by enhancing the metabolic ability of neurons and stimulating anti-inflammatory, anti-apoptosis and anti-oxidation responses. It is also attracting attention for its possible role in diseases such as stroke, brain injury and depression (Salehpour et al., 2018). In a randomized controlled clinical study, Tomazoni et al. (2019) found that pre-exercise PBMT has an important antioxidant effect, reducing exercise-induced oxidative stress. Besides, in the diabetic rat model, 670 nm PBM could protect liver by attenuating OS and enhancing the antioxidant protection. In this study, we found liver glutathione reductase and superoxide dismutase activity returned to normal, and glutathione peroxidase and glutathione S-transferase activity significantly increased in acute diabetic rats treated with light therapy (Lim et al., 2009). Low-level light therapy (LLLT) is a type of PBMT. The absorption of red/NIR light energy could enhance mitochondrial ATP production and attenuates oxidative stress without inciting tissue injury, photothermal or photoacoustic effect (Karu, 1999; Yamada et al., 2020). At present, LLLT is mainly used for diseases of body surface in clinic, such as facial rhytids, androgenic alopecia, acne vulgaris wound healing and et al. (Glass, 2021). Besides, LLLT is also applied for cancers and bone-related disorders (Mansouri et al., 2020). At present, there are no studies on the use of PBMT and LLLT in HIRI treatment. In theory, however, PBMT and LLLT have the potential to be therapeutic tools for HIRI because of their ability to inhibit oxidative stress. This becomes a research direction for us in the future.

## 5 Conclusion

In recent years, the mechanisms of HIRI and approaches to its diagnosis and prevention have been the focus of researchers. As one of the main mechanisms of HIRI, the research on OS would provide direction for the disgnosis and protection of hepatic ischemia-reperfusion injury. In basic research, optical imaging can monitor the ROS fluctuation during HIRI, which may be helpful for the study of specific signal pathways. In clinical, optical imaging technologies for oxidative stress may provide real-time, effective and non-invasive diagnosis for clinical workers in the future. However, there is a large gap between the exploration of animal models and the actual clinical use of these optical sensors in patients. Despite a large number of preclinical studies on optical imaging techniques for the diagnosis of HIRI, the results of their clinical trials are not encouraging. To date, although very few optical imaging products have been approved for the diagnosis of liver IRI, the optical imaging is still the most potencial tool to apply in diagnosis and monitering of HIRI. Therefore, in the future, we should also focus on how to promote clinical trials. The tissue penetration, mass production, storage stability, safety and non-toxicity of optical sensors should be considered as priorities for their successful clinical application in the diagnosis of HIRI and need to be further improved in future optical medicine-related research. And, it should also be noted that near-infrared light may induce thermal or phototoxic damages. When optical imaging is researched to be used *in vivo* clinical diagnosis, attention should be paid to avoid this problem. In conclusion, our future focus should be on *in vivo* studies and clinical trials using these optical imaging techniques to accelerate clinical transformation, which will have great significance in the diagnosis of HIRI.

Light therapies such as PDT, PTT, PBMT, and LILT have been used for cancers and other diseases. Moreover, PBMT and LILT have been shown to inhibit oxidative stress. Theoretically, light therapy for ROS may also have a protective effect in HIRI. Recent advances in the role of PBMT and LILT in anti-oxidative stress provide an intriguing new potential therapy for HIRI. So far, however, PBMT has not been applied in HIRI, and the protective effect of LILT on HIRI remains at the laboratory level. The application of PBMT and LILT in HIRI is just a prospect. Therefore, we think more attention should be paid to the further study on the mechanism of anti-oxidative stress in PBMT and LILT, and to confirm their effectiveness in HIRI management.
